# Post-treatment three-dimensional voxel-based dosimetry after Yttrium-90 resin microsphere radioembolization in HCC

**DOI:** 10.1186/s13550-022-00879-x

**Published:** 2022-02-04

**Authors:** Emile B. Veenstra, Simeon J. S. Ruiter, Robbert J. de Haas, Reinoud P. H. Bokkers, Koert P. de Jong, Walter Noordzij

**Affiliations:** 1 Department of Nuclear Medicine and Molecular Imaging, Medical Imaging Center, University Medical Center Groningen, University of Groningen, P.O. Box 30.001, 9700 RB Groningen, The Netherlands; 2 Department of Hepato-Pancreato-Biliary Surgery and Liver Transplantation, University Medical Center Groningen, University of Groningen, Groningen, The Netherlands; 3 Department of Radiology, Medical Imaging Center, University Medical Center Groningen, University of Groningen, Groningen, The Netherlands

**Keywords:** Dosimetry, Selective internal radiation therapy, Hepatocellular carcinoma, Dose-volume histogram, Tumour-absorbed dose, Dose–response effects

## Abstract

**Background:**

Post-therapy [^90^Y] PET/CT-based dosimetry is currently recommended to validate treatment planning as [^99m^Tc] MAA SPECT/CT is often a poor predictor of subsequent actual [^90^Y] absorbed dose. Treatment planning software became available allowing 3D voxel dosimetry offering tumour-absorbed dose distributions and dose-volume histograms (DVH). We aim to assess dose–response effects in post-therapy [^90^Y] PET/CT dosimetry in SIRT-treated HCC patients for predicting overall and progression-free survival (OS and PFS) and four-month follow-up tumour response (mRECIST). Tumour-absorbed dose and mean percentage of the tumour volume (V) receiving ≥ 100, 150, 200, or 250 Gy and mean minimum absorbed dose (D) delivered to 30%, 50%, 70%, and 90% of tumour volume were calculated from DVH’s. Depending on the mean tumour -absorbed dose, treated lesions were assigned to a < 120 Gy or ≥ 120 Gy group.

**Results:**

Thirty patients received 36 SIRT treatments, totalling 43 lesions. Median tumour-absorbed dose was significantly different between the ≥ 120 Gy (*n* = 28, 207 Gy, IQR 154–311 Gy) and < 120 Gy group (*n* = 15, 62 Gy, IQR 49–97 Gy, *p* <0 .01). Disease control (DC) was found more frequently in the ≥ 120 Gy group (79%) compared to < 120 Gy (53%). Mean tumour-absorbed dose optimal cut-off predicting DC was 131 Gy. Tumour control probability was 54% (95% CI 52–54%) for a mean tumour-absorbed dose of 120 Gy and 90% (95% CI 87–92%) for 284 Gy. Only D30 was significantly different between DC and progressive disease (*p* = 0.04). For the ≥ 120 Gy group, median OS and PFS were longer (median OS 33 months, [range 8–33 months] and median PFS 23 months [range 4–33 months]) than the < 120 Gy group (median OS 17 months, [range 5–33 months] and median PFS 13 months [range 1–33 months]) (*p* < 0.01 and *p* = 0.03, respectively).

**Conclusions:**

Higher 3D voxel-based tumour-absorbed dose in patients with HCC is associated with four-month DC and longer OS and PFS. DVHs in [^90^Y] SIRT could play a role in evaluative dosimetry.

## Background

Selective internal radiation therapy (SIRT) has been established as a form of treatment for non-operable and locally advanced hepatocellular carcinoma (HCC) in the liver [1, 2]. Both glass (TheraSphere®, Boston Scientific Corporation, Marlborough, MA, USA) and resin microspheres with yttrium-90 (SIR-Sphere®, Sirtex Medical Limited Australia, Sydney, Australia) are commonly used.

During treatment planning, a diagnostic liver angiography is performed with intra-arterial injection of gamma-emitting ^99m^Tc-labelled macro-albumin aggregates ([^99m^Tc] MAA) at the proposed arterial treatment position. This is followed by perfusion scintigraphy (SPECT/CT) to determine potential hepatopulmonary shunting and extrahepatic distribution.

Pre-operative dosimetry is used to personalize [^90^Y] dosage and predict whether there will be sufficient accumulation of beta-emitting ^90^Y-microspheres in the target tumours. For SIRT, dosimetry based on ^99m^Tc-MAA SPECT/CT prior to treatment, or a direct ^90^Y PET/CT quantification after treatment are available. Two dosimetry methods are recommended to calculate appropriate injected 90Y-activity for resin microspheres: body surface area (BSA) and partition model method [3]. Both methods assume homogeneity of tissue or resin distribution, limiting their objectivity. Recently, treatment planning software became available allowing 3D dosimetry at voxel level. Voxel-based dosimetry allows 3D visualization of tumour-absorbed dose distributions and evaluation of degree of heterogeneity through dose-volume histograms (DVH) [4, 5].

Tumour response and clinical outcomes of HCC following SIRT vary considerably, ranging from no response in certain patients to excellent results in others [6]. Large phase II trials, however, found no overall survival benefit [7, 8]. Potentially, this could be caused by the investigated dose–response relationships. Dose–response relationships have been demonstrated for resin microspheres [6, 9–12], resulting in tumour dose–response thresholds between 100 and 120 Gy [13], which is the current recommendation for resin microspheres [4].

Post-therapy [^90^Y] PET/CT-based dosimetry can validate treatment delivery as [^99m^Tc]MAA SPECT/CT is often a poor predictor of subsequent actual ^90^Y absorbed dose [14]. SIRT treatment verification and dosimetry with [^90^Y] PET/CT are currently recommended [4]. We hypothesize that ^90^Y PET/CT-based dosimetry predicts better treatment responses in lesions receiving more than 120 Gy tumour-absorbed dose compared to less than 120 Gy. Therefore, the aim of this study is to assess post-therapy dosimetry between SIRT-treated HCC patients with lesions receiving more or less than 120 Gy tumour-absorbed dose and mRECIST observed responses.

## Methods

Patients with unresectable HCC treated with [^90^Y] resin microspheres SIRT in our institution from May 2018 to November 2020 were considered for this retrospective study. Inclusion criteria consisted of contrast-enhanced CT or MRI which was performed 12 weeks prior to SIRT, a targeted lesion long-axis diameter of at least 2 cm, and a follow-up MRI at four months. Only patients receiving Sirtex ^90^Y-resin microspheres were included, as resin and glass microspheres differ in general kinetics and dose calculation. Individual informed consent was not required, because studies involving a retrospective review, collection, and analysis of patient records do not fall under the scope of the Dutch Act on Medical Scientific Research involving Human Beings (WMO). For privacy, data were stored and analysed anonymously. Patient characteristics, such as age, sex, comorbidities, other risk factors and outcomes, were extracted from the electronic medical records. Overall survival (OS) and progression-free survival (PFS) were noted. Relevant follow-up therapy and staging of Barcelona-Clinic Liver Cancer (BCLC) and Child–Pugh (CP) were noted. Evaluation of treatment response to SIRT was done according to the modified response evaluation criteria (mRECIST) at 4-month MRI [15, 16].

### Planning angiography and ^99m^Tc‑MAA SPECT/CT

All patients were subjected to angiography of the upper abdominal vessels to define vascular anatomy and to assess optimal catheter-tip placement [4]. Following angiography, 150 MBq (4 mCi) of [^99m^Tc] MAA (Pulmocis, Curium Pharma, Petten, the Netherlands) was administered. One hour after injection of [^99m^Tc] MAA, lung and liver planar scan and low dose, no contrast-enhanced SPECT/CT acquisitions were performed using a hybrid scanner combining a dual-head gamma camera and a 2**-**slice SPECT/CT scanner (Symbia T2, Siemens Healthcare, Germany). Images were then reconstructed on a Siemens workstation (SyngoVia VB30, Siemens Healthcare, Germany). The amount of ^90^Y-microsphere activity needed during treatment phase was determined by the partition model, provided and detailed by the manufacturer (SIR-Sphere®, Sirtex Medical Limited Australia, Sydney, Australia) [4, 15].

### SIRT and [^90^Y] PET/CT

SIRT was performed within two weeks after planning angiography. The planned activity of ^90^Y-loaded microspheres was injected through a microcatheter at the same position as determined during planning angiography. Within one day after SIRT, patients underwent [^90^Y] PET/CT scan (Biograph mCT PET/CT, Siemens Healthcare, Erlangen, Germany) with a maximum of two bed positions, and 15-min acquisition per bed position, for treatment verification and post-treatment dosimetry. PET data were reconstructed with Siemens Ultra HD (TrueX and time of flight), using three iterations and 21 subsets with a 400-matrix size and a 9-mm Gaussian (isotropic) filter. Attenuation and scatter correction of PET emission data were achieved by a low-dose CT scan with 120 kV and 35 mAs.

### Dosimetry

For pre-treatment planning of injected ^90^Y-activity, liver and tumour contours were manually delineated on CT images, acquired during planning angiography to be used in the partition model. Pre-treatment contrast-enhanced CT (Siemens SOMATOM Force CT) or gadolinium-enhanced fat-saturated T1-weighted MRI (Siemens Magnetom Skyra MRI) were used for 3D delineation of liver and tumour contours for post-treatment dosimetry. Post-treatment dosimetry contouring was performed in MIM SurePlan (v7.0.4, MIM software, Cleveland, USA). In all three planes and for every three slides, the researcher manually delineated vital liver tissue and tumours. The software then interpolated all contours to create a 3D representation of all contours. These contours were then transformed to contours on post-therapy PET/CT by a MIM SurePlan clinical workflow (“90Y Dose Calculation”) using deformable registration algorithms. The computed contours were then, in some cases, manually translated or rotated to achieve optimum visual fit.

^90^Y-dose and DVH for each tumour were calculated with the local deposition method (LDM), as previously described [16]. The mean tumour-absorbed dose (in Gy) were extracted from DVH, where area under the DVH (AUDVH) equals tumour-absorbed dose [17]. V100, V150, V200, and V250 were calculated from the DVH, representing the percentage of the tumour volume receiving indicated value of radiation (in Gy). D30, D50, D70, and D90 were computed showing the minimum absorbed dose delivered to those tumour volume percentages.

Excluding small tumours reduced the chance of partial volume effects of dosimetry data in relation to the PET/CT, as a sphere diameter of at least 2 cm with no filtering should give a better reading of activity according to the literature [18]. Depending on the mean tumour-absorbed dose, treated lesions were assigned to a < 120 Gy or ≥ 120 Gy group. Patients who had both a < 120 Gy and a ≥ 120 Gy lesion were added to both groups. For computing OS, time between first (or only) treatment and death was calculated and, patients were not added twice in case of group comparisons. Complete response (CR), partial response (PR), and stable disease (SD) mRECIST results were combined into a disease control (DC) group to be compared to progressive disease (PD).

### Statistics

All descriptive statistics are given by numbers with percentiles or the median with its interquartile ranges, unless stated otherwise. Comparisons of tumour-absorbed dose between DC and PD are performed by an unpaired t test with Welch’s correction. Comparisons of mRECIST with mean tumour dose and D- and V-values were compared by Kruskal–Wallis (with Dunn’s multiple comparisons test) or two-way ANOVA. A nonlinear second-order polynomial (quadratic) least squares fit was performed on the DVH of DC and PD groups. Receiver operating characteristic (ROC) analysis was performed to identify the optimal cut-off (defined by the Youden index) of tumour-absorbed dose to predict DC. By averaging the chance of all patients to have DC, binned by intervals of 20 Gy, the tumour control probability (TCP) was computed and related to tumour dose using a linear quadratic model. OS and PFS between-group comparisons were determined with Kaplan–Meier Chi-square log rank Mantel–Cox. Statistical analysis was performed using SPSS version 23.0 software (SPSS Inc., Chicago, IL). *p* values lower than 0.05 were considered to be significant.

## Results

**Fig. 1:**
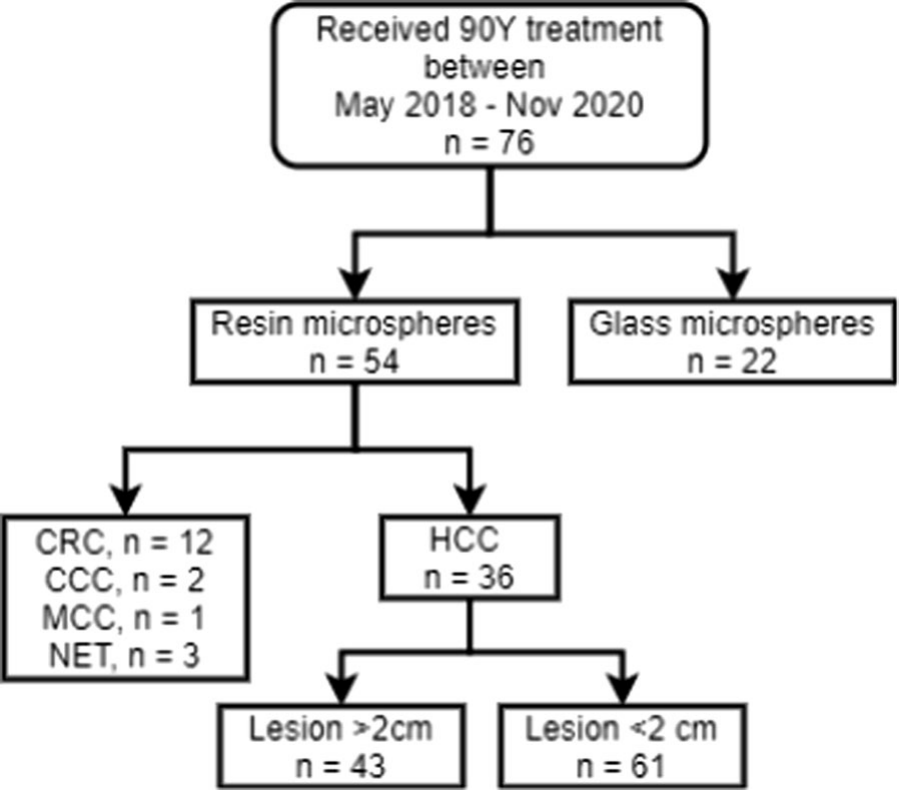
Flowchart for the selection of patients. *CRC* colorectal cancer, *CCC* cholangiocellular carcinoma, *MBC* metastatic breast cancer, *NET* neuroendocrine tumour

Thirty patients (26 male, 4 female) with unresectable HCC underwent [^90^Y] SIRT resin microspheres treatments and subsequent post-therapy [^90^Y] PET/CT scanning in our institution between May 2018 and November 2020. A total of 36 treatments were performed, as six patients were treated two times. A total of 104 lesions were found, of which 43 lesions could be included (Fig. 1). Patient characteristics are detailed in Table 1. Patient characteristics did not differ between both groups. For pre-SIRT liver and tumour contouring, appropriate CT and MR images were available in 11 and 25 treatments, respectively.

**Table 1:** Patient characteristics

	All patients (*n* = 30)
Median age (y, IQR)	70 (63–73)
Sex	
M	26
F	4
BCLC staging (per treatment *n* = 36)	
A	13
B	20
C	3
Child–Pugh (per treatment *n* = 36)	
0–1–2	2
5	22
6	10
7	2
Pre-SIRT treatment received	
None	21
SIRT	5
Resection	4
RFA	4
TACE	2
SABR	1
Reoccurrence location	
SIRT-residue	27
None	3
Liver, Non-SIRT	13
Extrahepatic metastasis	3
Follow-up	
Alive, no recurrence	5
Alive, with recurrence	15
Died	10

*IQR* interquartile range, *BCLC* Barcelona clinic liver cancer, *SIRT* selective internal radiation therapy, *RFA* radiofrequency ablation, *TACE* transarterial chemoembolization, *SABR* stereotactic ablative body radiotherapy

### SIRT and dose analysis

Right liver hemisphere SIRT was predominantly performed (20/36), followed by left (11), whole (4) and segmental (1; Table 2). One radiation-related complication occurred involving gastric radiation exposure. The median administered activity was 1.18 GBq (range 0.25–1.94 GBq). Median ^90^Y-injected activity was not significantly different between the ≥ 120 Gy (1435 MBq, IQR: 873–1550) and the < 120 Gy group (1000 MBq, IQR: 600–1300, *p* = 0.08). Median tumour-absorbed dose was significantly lower in the < 120 Gy group (62 Gy, IQR: 49–97 Gy), compared to ≥ 120 Gy (207 Gy, IQR: 154–311 Gy, *p* < 0.01; Table 3). Median vital liver dose was equal between the < 120 Gy (23 Gy, IQR: 9–26 Gy) and ≥ 120 Gy group (23 Gy, IQR: 14–28 Gy; *p* = 0.51).

**Table 2:** SIRT characteristics

	Per treatment (*n* = 36)	Per lesion (*n* = 43)	
		< 120 Gy (*n* = 15)	≥ 120 Gy (*n* = 28)
SIRT localization (Mean ^90^Y-injected)			
Right	20 (56%) (1148 MBq)	12 (80%)	13 (46%)
Left	11 (31%) (901 MBq)	2 (13%)	9 (32%)
Whole	4 (11%) (1675 MBq)	1 (6%)	5 (18%)
Segmental	1 (3%) (1940 MBq)	0	1 (4%)
Complications			
Moderate	1 (3%)		
Tumour number			
Single	12 (33%)		
Multiple	24 (67%)		
Tumour response (4-month mRECIST)			
Complete response	3 (83%)	2 (13%)	2 (7%)
Partial response	14 (39%)	4 (27%)	15 (54%)
Stable disease	6 (17%)	2 (13%)	5 (18%)
Progressive disease	13 (36%)	7 (47%)	6 (21%)
Objective response^†^	17 (53%)	6 (40%)	17 (61%)
Disease control^††^	23 (67%)	8 (53%)	22 (79%)

*SIRT* selective internal radiation therapy, *MBq* megabecquerel, *Gy* Gray, *mRECIST* modified response evaluation criteria in solid tumours

^†^Objective response is the proportion of treatment sessions or lesions with complete or partial response

^††^Disease control is the proportion of treatment sessions or lesions with complete or partial response or stable disease

**Table 3:** Dosimetry characteristics

	Amount (*n*)	^90^Y-injected Dose^†^ (MBq, median, IQR)	Tumour-absorbed dose (Gy, median, IQR)	Radiation-absorbed vital liver dose (Gy, median, IQR)
< 120 Gy	15	1000 (600–1300)	62 (49–97)	23 (9–26)
≥ 120 Gy	28	1435 (873–1550)	207 (154–311)	23 (14–28)
		(*p* = 0.08)	(*p* < 0.01)	(*p* = 0.51)

*Gy* Gray, *MBq* megabecquerel, *IQR* interquartile range

^†^In case of whole liver SIRT, only the amount of ^90^Y directed to the side of the measured lesion was included

### Dose-volume response analysis

**Fig. 2:**
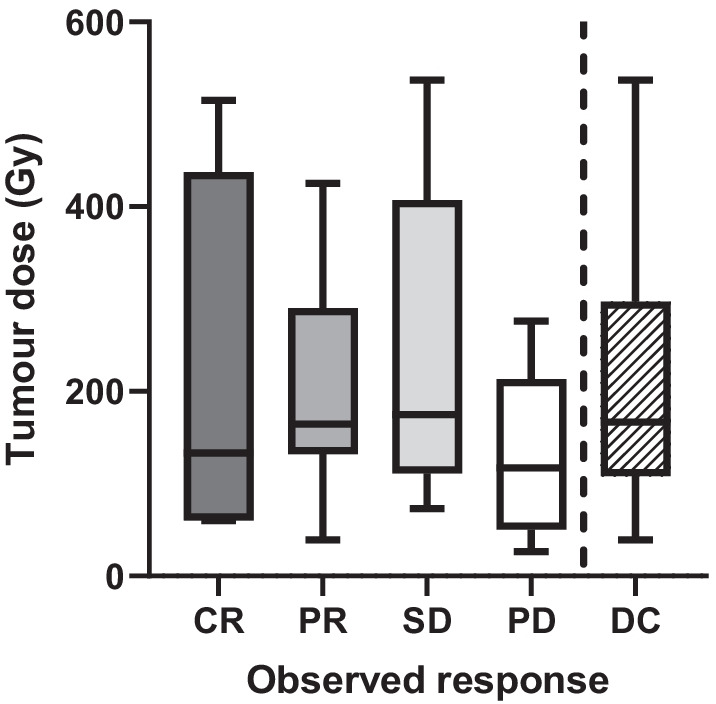
Mean tumour dose for observed responses (mRECIST). Group comparisons showed no significant differences. *CR* complete response, *PR* partial response, *SD* stable disease, *PD* progressive disease, *DC* disease control

**Fig. 3:**
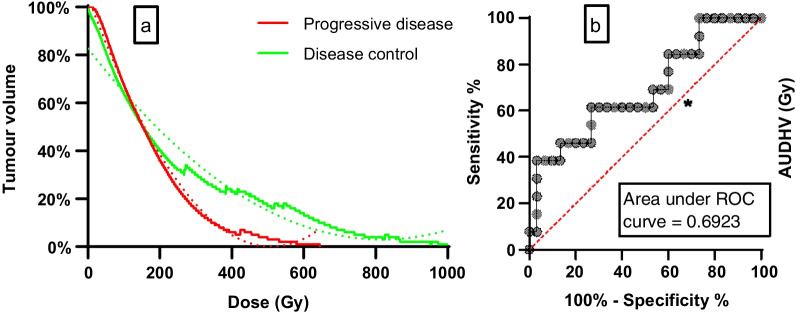
**a** AUDVH of lesions showing DC (*n* = 30) and PD (*n* = 13), where AUDVH represents tumour-absorbed dose (*p* = 0.05). **b** ROC analysis for AUDVH to predict DC. Optimal cut-off noted with asterisk at 131 Gy, with 61.54% sensitivity and 73.33% specificity). The area under the ROC curve of AUDVH for predicting DC was 0.692 (95% CI 0.515–0.869). *AUDVH* area under dose-volume histogram, *DC* disease control, *PD* progressive disease

**Fig. 4:**
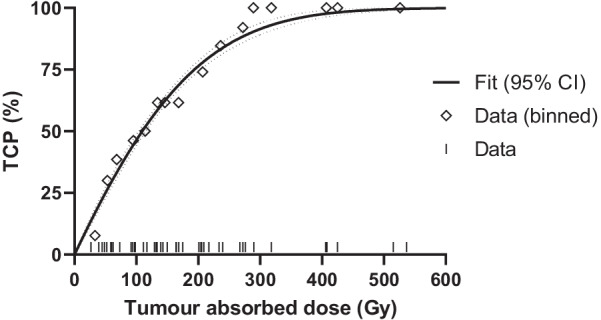
Tumour control probability (TCP) plotted in relation to tumour-absorbed dose. Black curve is a fit of linear quadratic model (95% confidence intervals dotted in grey). Binned data with 20 Gy intervals are shown with diamond shapes. Individual cases are represented by black lines in the bottom. *CI* confidence interval

**Fig. 5:**
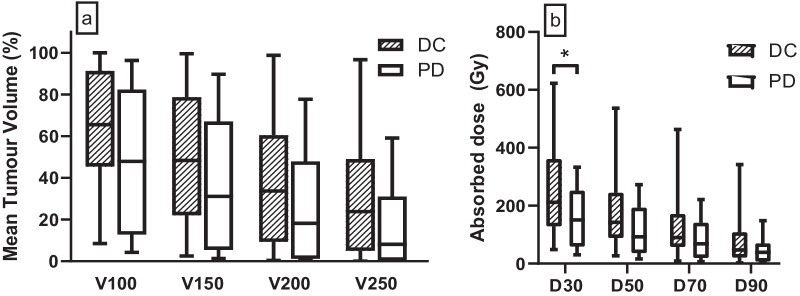
**a** Mean tumour volume percentage (V) at V100, V150, V200, and V250. **b** Mean minimum absorbed dose (D) delivered for D30 (*p* = 0.04), D50, D70, and D90. *DC* disease control, *PD* progressive disease

The four-month DC rate was 70% (*n* = 30/43), including four cases of CR, 19 cases with PR, and seven cases with SD. DC and objective response rates were higher in the ≥ 120 Gy group (79% and 53%) compared to < 120 Gy (53% and 40%, Table 2). Mean tumour-absorbed dose for each observed response were not significantly different between each other (Fig. 2). The optimal tumour-absorbed dose cut-off for predicting DC was 131 Gy, resulting in 62% sensitivity and 73% specificity (Fig. 3c). TCP was 54% (95% CI 52–54%) for a mean tumour-absorbed dose of 120 Gy and 90% (95% CI 87–92%) for 284 Gy (Fig. 4). Percentage of tumour receiving ≥ 100, 150, 200, or 250 Gy (V100, V150, V200, and V250; Fig. 5a) was not significantly different between DC and PD. Only D30 was significantly different between DC and PD (*p* = 0.04, Fig. 5b).

### Dose-survival analysis

**Fig. 6:**
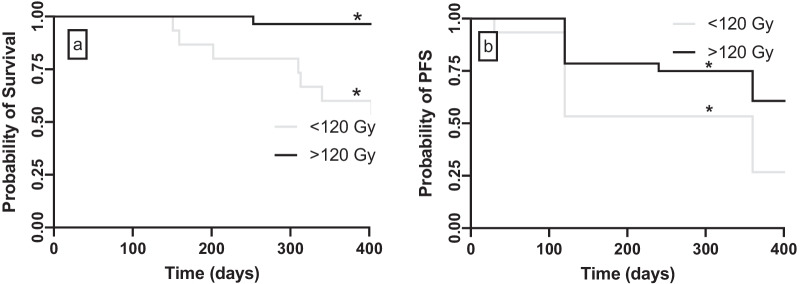
**a** Kaplan–Meier overall survival (OS) estimates (*p* < 0.01), **b** Kaplan Meier progression-free survival (PFS) estimates (*p* = 0.03)

Median follow-up for OS and PFS were 27 months (range 10–40 months). For the ≥ 120 Gy group, median OS and PFS were longer (median OS 33 months, [range 8–33 months] and median PFS 23 months [range 4–33 months]) than the < 120 Gy group (median OS 17 months, [range 5–33 months] and median PFS 13 months [range 1–33 months]) (*p* < 0.01 and *p* = 0.03, respectively; Fig. 6a and 6b). Ten patients died following SIRT with a median of 312 days (IQR: 206–317 days), of which nine < 120 Gy. One patient with a single ≥ 120 Gy lesion died during follow-up. All deaths in our study occurred due to progression of liver disease.

## Discussion

We aimed to examine mRECIST observed responses in ≥ 120 Gy lesions compared to < 120 Gy by post-therapy dosimetry. We found equal injected ^90^Y-dose between cases receiving more than ≥ 120 Gy or less, while mean tumour doses were widespread in both groups. This demonstrates that the planned and actual tumour dose can be considerably different and confirms the need for quantitative dose–response analysis by the use of post-therapy [^90^Y] PET/CT in the treatment of locally advanced HCC. Patients with lesions receiving more than ≥ 120 Gy showed longer overall and progression free survival.

Generally, mean tumour dose can be used to determine ^90^Y-SIRT efficacy [19]. As it assumes uniform dose distribution, much attention has been given to the analysis of DVHs. The introduction of tumour-absorbed dose, D70 (minimum absorbed dose delivered to 70% of the tumour), and V100 (percentage of the tumour volume receiving ≥ 100 Gy) may further increase the understanding of actual heterogeneous deposition of 90Y microspheres. Both D70 and V100 were determined by a study with six patients in which these specific values acted as a threshold for complete versus incomplete responses [14]. We explored several ranges of D(30–90%) and V(100–250 Gy) showing that there is a non-significant trend of PD always scoring lower to DC. Further investigation of the clinical relevance of these values is needed.

Several studies on post-therapy [^90^Y] PET/CT in HCC with resin microspheres have been performed [4]. A study with 43 SIRT procedures found that tumour AUDVH was associated with DC, with an optimal cut-off of 61 Gy (76% sensitivity and specificity) [6]. Another study with 73 participants reported 50% TCP at 110–120 Gy [12]. Several case studies validated the feasibility of post-therapy PET with [^90^Y] SIRT, with one study finding a tumour-absorbed dose of 287 Gy showing complete remission after 6 month [20, 21]. Another study suggested a relationship between higher [^90^Y] dose and better tumour response, noting that treatment responders had a mean tumour-absorbed dose of 215 Gy [22]. We found that AUDVH, and thereby tumour-absorbed dose, was associated with DC. DC was optimally predicted by a mean tumour dose of 131 Gy, and 50% TCP was achieved at 110 Gy. Non-conformity of our results to previous literature could be due to exclusion of lesions smaller than two cm and because a third of all included lesions were solitary HCC tumours. Both can lead to finding higher tumour dosages. Considering all available literature and our results, a potential trend between higher tumour doses than 120 Gy and better tumour response is likely. Currently, no standard exists for [^90^Y] DVH reporting, and comparisons between studies are difficult as a result of different dose calculation methods, response evaluations, and low number of patients [23].

Current international recommendations determine a mean tumour-absorbed dose of 100 to 120 Gy for HCC [4]. In the present study, the achieved mean tumour-absorbed dose of 215 Gy in DC compared to 134 Gy in the PD group using the recommended dosimetry and administration protocols aligned with these suggested thresholds. Individual examination of the included tumour showed a wide variation of tumour doses. It has been proposed that a higher dose can not only target the primary tumour more effectively, but can also lead to the targeting of, often undetected, small satellite lesions [19]. In our study, post-SIRT novel lesions were seen in seven out of 13 PD cases. Further examinations of tumour-absorbed dose, volume over time and lesion-based response evaluations are in progress. We found that higher tumour-absorbed doses were well-tolerated, as vital liver dose was not different between groups and only one moderately severe radiation-related complication occurred.

Limitations of our study include its retrospective design and low number of patients, although patient characteristics were generally uniform. Dose prediction based on [^99m^Tc]MAA SPECT was not within the scope of this study, as its predictive value for delivered dose is still subject of debate in the literature [24]. As a result of our lesion-level analysis, several included lesions came from the same patients. No lesions were included that were targeted by a previous bout of SIRT, but we cannot rule out any second-degree radiation effects due to the lesion being part of the same treated liver hemisphere. We found that one patient had pre-therapy CT/MRI 9 weeks before SIRT and although no apparent changes in tumour presentation were noted, no objective measures were taken to control for lesion size changes during this period. For all patients, four-month post-SIRT MRI were retrieved, but later MRI follow-up were sporadic and only survival characteristics could be accurately determined after four months. By excluding lesions smaller than 2 cm, we aimed to reduced partial volume effects, such as breathing and resolution artefacts, of PET data. Nevertheless, this led to some cases of mismatch between pre- and post-therapy contours. These contours needed manual alterations to achieve optimal fit, which might lead to overestimations of tumour-absorbed dose.

## Conclusion

Resin-microsphere SIRT with post-therapy voxel-based mean tumour-absorbed doses above 120 Gy in patients with HCC is associated with four-month DC and longer OS and PFS. DVHs in [^90^Y] SIRT could play a role in evaluative dosimetry. These results demonstrate the need for further validation of optimal tumour dose and dose distribution characteristics with post-therapy [^90^Y] PET/CT dosimetry.

## Data Availability

The datasets used and/or analysed during the current study are available from the corresponding author on reasonable request.

## Abbreviations

DVH: Dose-volume histograms
AUDVH: Area under dose-volume histogram
SIRT: Selective internal radiation therapy
OS: Overall survival
PFS: Progression-free survival
Vx: Mean percentage of the tumour volume receiving x Gy
Dx: Mean minimum absorbed dose delivered to x of tumour volume
99mTc-MAA: 99MTc-labelled macro-albumin aggregates
HCC: Hepatocellular carcinoma
BCLC: Barcelona-Clinic Liver Cancer
CP: Child–Pugh
mRECIST: Modified response evaluation criteria
CR: Complete response
PR: Partial response
SD: Stable disease
DC: Disease control
PD: Progressive disease
ROC: Receiver operating characteristic
TCP: Tumour control probability

## References

[1] Benson AB, D’Angelica MI, Abbott DE, Abrams TA, Alberts SR, Anaya DA (2019). Hepatobiliary cancers, version 2.2019 featured updates to the NCCN guidelines. J Natl Compr Cancer Netw..

[2] Vogel A, Cervantes A, Chau I, Daniele B, Llovet J, Meyer T (2018). Hepatocellular carcinoma: ESMO Clinical Practice Guidelines for diagnosis, treatment and follow-up. Ann Oncol..

[3] Lau WY, Kennedy AS, Kim YH, Lai HK, Lee RC, Leung TWT (2012). Patient selection and activity planning guide for selective internal radiotherapy with yttrium-90 resin microspheres. Int J Radiat Oncol Biol Phys.

[4] Levillain H, Bagni O, Deroose CM, Dieudonné A, Gnesin S, Grosser OS (2021). International recommendations for personalised selective internal radiation therapy of primary and metastatic liver diseases with yttrium-90 resin microspheres. Eur J Nucl Med Mol Imaging.

[5] Cremonesi M, Ferrari ME, Bodei L, Chiesa C, Sarnelli A, Garibaldi C (2018). Correlation of dose with toxicity and tumour response to 90 Y- and 177 Lu-PRRT provides the basis for optimization through individualized treatment planning. Eur J Nucl Med Mol Imaging.

[6] Allimant C, Kafrouni M, Delicque J, Ilonca D, Cassinotto C, Assenat E (2018). Tumor targeting and three-dimensional voxel-based dosimetry to predict tumor response, toxicity, and survival after Yttrium-90 resin microsphere radioembolization in hepatocellular carcinoma. J Vasc Interv Radiol.

[7] Chow PKH, Gandhi M, Tan S-B, Khin MW, Khasbazar A, Ong J (2018). SIRveNIB: selective internal radiation therapy versus sorafenib in Asia-pacific patients with hepatocellular carcinoma. J Clin Oncol.

[8] Vilgrain V, Pereira H, Assenat E, Guiu B, Ilonca AD, Pageaux GP (2017). Efficacy and safety of selective internal radiotherapy with yttrium-90 resin microspheres compared with sorafenib in locally advanced and inoperable hepatocellular carcinoma (SARAH): an open-label randomised controlled phase 3 trial. Lancet Oncol.

[9] Lau WY, Leung WT, Ho S, Leung NWY, Chan M, Lin J (1994). Treatment of inoperable hepatocellular carcinoma with intrahepatic arterial yttrium-90 microspheres: a phase I and II study. Br J Cancer.

[10] Hermann A-L, Dieudonné A, Maxime R, Manuel S, Helena P, Gilles C (2018). Role of 99mTc-macroaggregated albumin SPECT/CT based dosimetry in predicting survival and tumor response of patients with locally advanced and inoperable hepatocellular carcinoma (HCC) treated by selective intra-arterial radiation therapy (SIRT) with yttrium-90 resin microspheres, a cohort from SARAH study. J Hepatol.

[11] Kao YH, Tan AEH, Burgmans MC, Irani FG, Khoo LS, Lo RHG (2012). Image-guided personalized predictive dosimetry by artery-specific SPECT/CT partition modeling for safe and effective 90Y radioembolization. J Nucl Med.

[12] Strigari L, Sciuto R, Rea S, Carpanese L, Pizzi G, Soriani A (2010). Efficacy and toxicity related to treatment of hepatocellular carcinoma with 90Y-SIR spheres: radiobiologic considerations. J Nucl Med.

[13] Garin E, Palard X, Rolland Y (2020). Personalised dosimetry in radioembolisation for HCC: impact on clinical outcome and on trial design. Cancers (Basel).

[14] Kao YH, Steinberg JD, Tay YS, Lim GK, Yan J, Townsend DW (2013). Post-radioembolization yttrium-90 PET/CT-part 2: dose–response and tumor predictive dosimetry for resin microspheres. EJNMMI Res.

[15] Sirtex. SIR-Spheres ® microspheres [Internet]. 2020. Cited 6 Apr 2021. p. 1–84. http://www.sirtex.com/media/29845/ssl-us-10.pdf.

[16] Chiesa C, Mira M, Maccauro M, Spreafico C, Romito R, Morosi C (2015). Radioembolization of hepatocarcinoma with 90Y glass microspheres: development of an individualized treatment planning strategy based on dosimetry and radiobiology. Eur J Nucl Med Mol Imaging.

[17] Walrand S, Chiesa C, Gabina PM, Chouin N, Gear J, Stokke C (2019). Re: Tumor targeting and three-dimensional voxel-based dosimetry to predict tumor response, toxicity, and survival after yttrium-90 resin microsphere radioembolization in hepatocellular carcinoma. J Vasc Interv Radiol.

[18] Soret M, Bacharach SL, Buvat I (2007). Partial-volume effect in PET tumor imaging. J Nucl Med.

[19] Cremonesi M, Chiesa C, Strigari L, Ferrari M, Botta F, Guerriero F (2014). Radioembolization of hepatic lesions from a radiobiology and dosimetric perspective. Front Oncol.

[20] D’Arienzo M, Filippi L, Chiaramida P, Chiacchiararelli L, Cianni R, Salvatori R (2013). Absorbed dose to lesion and clinical outcome after liver radioembolization with 90Y microspheres: a case report of PET-based dosimetry. Ann Nucl Med.

[21] Lhommel R, Van Elmbt L, Goffette P, Van Den Eynde M, Jamar F, Pauwels S (2010). Feasibility of 90Y TOF PET-based dosimetry in liver metastasis therapy using SIR-Spheres. Eur J Nucl Med Mol Imaging.

[22] Srinivas SM, Natarajan N, Kuroiwa J, Gallagher S, Nasr E, Shah SN (2014). Determination of radiation absorbed dose to primary liver tumors and normal liver tissue using post-radioembolization 90Y PET. Front Oncol..

[23] Lea WB, Tapp KN, Tann M, Hutchins GD, Fletcher JW, Johnson MS (2014). Microsphere localization and dose quantification using positron emission tomography/ct following hepatic intraarterial radioembolization with yttrium-90 in patients with advanced hepatocellular carcinoma. J Vasc Interv Radiol.

[24] Garin E, Rolland Y, Laffont S, Edeline J (2016). Clinical impact of 99mTc-MAA SPECT/CT-based dosimetry in the radioembolization of liver malignancies with 90Y-loaded microspheres. Eur J Nucl Med Mol Imaging.

